# Effect of cigarette smoke on surface roughness and bacterial adhesion of orthodontic aligners: an in vitro investigation

**DOI:** 10.1007/s00784-025-06720-8

**Published:** 2026-01-17

**Authors:** Kelly Galisteu-Luiz, Flávio de Mendonça Copello, Karla Lorene de França Leite, Mylena da Rocha Cavalcante, Larine Ferreira Lira, Kenderson Santos Silva, Sarah Pereira Martins, Ingrid Cristina Pinto da Costa, Gabriela Drago Vidal, Carlos Nelson Elias, Luísa Schubach da Costa Barreto, Margareth Maria Gomes de Souza

**Affiliations:** 1 Department of Orthodontics and Pediatric Dentistry, School of Dentistry, Universidade Federal do Rio de Janeiro (UFRJ), Rio de Janeiro, RJ Brazil; 2 Department of Orthodontics and Pediatric Dentistry, School of Dentistry, University of Maryland, Baltimore, Baltimore, MD USA; 3 Department of Biomaterials, Military Engineering Institute (IME), Rio de Janeiro, RJ Brazil; 4 Department of Social and Preventive Dentistry (PRECOM), School of Dentistry, Universidade do Estado do Rio de Janeiro (UERJ), Boulevard Vinte e Oito de Setembro, 157, 2nd floor, Vila Isabel, Rio de Janeiro, CEP 20.551-030 RJ Brazil

**Keywords:** Orthodontic appliances, removable, Tobacco use, Surface properties, Bacteria, Dental plaque

## Abstract

**Objectives:**

This study aims to evaluate the impact of cigarette smoke on Invisalign^®^ aligners in surface roughness, bacterial adherence rates, pH changes and multispecies biofilm formation after exposure.

**Materials and Methods:**

Seventy-two flat samples of growth (G1), sterility (G2), saliva (G3), and smoke (G4) groups were exposed to artificial saliva, and G4 underwent 21 cycles of smoke. Linear roughness (Ra) and average depth (Rz) were assessed before and after saliva (G3.1) and smoke (G4.1) exposure. Independent and paired t-tests compared measurements over 5 days of evaluation [biofilm, microorganisms (CFU/mL), pH, and volumetric surface roughness (Sa)], measurements were taken before (T0) and after (T1) exposure to saliva and cigarette smoke.

**Results:**

Increased roughness post-exposure to cigarette smoke Ra (p = 0.003) and Rz (p = 0.014) in G4.1 at T1 (p = 0.046), and higher roughness over time (Ra: 0.139 ± 0.007; Rz: 0.741 ± 0.026), unlike G3.1 (Ra: 0.122 ± 0.002; Rz: 0.616 ± 0.021). G1 had the highest CFU (8.57 × 108), significantly higher than the G4.2 (5.33 × 108) and G3.2 (2 × 108). From 24 to 96 h, pH in G4.2 (5.75) and G1 (4.45) showed slight decreases, G2 (6.76) and G3.2 (6.35) increased. G1 had the highest Sa (48.97 ± 14.19), compared to G2 (4.27 ± 1.14), G3 (7.60 ± 4.28), and G4 (13.98 ± 8.18).

**Conclusions:**

Cigarette smoke exposure elevated surface roughness and enhanced bacterial adherence on aligners. Clinical Relevance: Reinforce smoking cessation and strict oral hygiene protocols for aligner-wearing patients. In vivo studies are warranted to confirm these findings.

## Introduction

Orthodontic aligners have become a favoured alternative to traditional braces, providing patients with a visually appealing and comfortable orthodontic treatment using thermoplastic materials derived from 3D virtual models [1]. This has contributed to their broad acceptance among both orthodontic professionals and patients. The growing popularity of aligner therapy has sparked the emergence of numerous transparent orthodontic aligner systems worldwide, following in the footsteps of Invisalign^®^ (Align Technology, Santa Clara, California, USA) [2]. This trend has notably increased the utilization of polymers in orthodontic treatment [3].

Transparent orthodontic aligners, such as Invisalign^®^, are primarily composed of advanced thermoplastic polymers, specifically a multilayer polyurethane and copolyester blend, which are engineered for durability, flexibility, and optical clarity [4]. When new, these aligners have a smooth, uniform surface that is designed to resist bacterial adhesion and provide comfort during wear [5]. However, due to the nature of their material, they remain susceptible to various environmental influences, including temperature fluctuations, moisture, salivary enzymes, and exposure to chemicals such as those found in cigarette smoke. These factors can gradually alter the surface properties of the aligners, particularly increasing surface roughness, which in turn creates a more favorable environment for microbial adherence and biofilm formation [6].

Smoking is a prevalent lifestyle habit among a considerable portion of the global adult population [7, 8]. As adults increasingly seek orthodontic treatment, particularly with a preference for more aesthetically pleasing options like transparent aligners [9], understanding the potential effects of cigarette smoke exposure on aligner surfaces becomes paramount. Previous studies have demonstrated that cigarette smoke can alter the surface roughness of aesthetic brackets [7] and orthodontic wires [10], raising questions about whether orthodontic aligners may also undergo surface modifications due to cigarette smoke exposure.

Changes in the surface roughness of polymer materials can facilitate greater bacterial biofilm adherence [11], particularly in patients who smoke, necessitating stricter biofilm control measures [12]. The adhesion of biofilm to orthodontic materials depends significantly on surface morphology [13], chemical nature [14], structure, and material properties [5]. Furthermore, there is a scarcity of literature on biofilm formation on aligner materials, despite biofilms being potential indicators of oral infections. Understanding the initiation, formation, and dissemination of biofilms on aligner materials is crucial for clinical management.

To date, no previous study has investigated the direct impact of cigarette smoke exposure on the surface roughness and subsequent biofilm formation on Invisalign^®^ aligners. Given these considerations, this study aims to evaluate possible alterations in the surface roughness, bacterial adherence rates, pH changes and multispecies biofilm formation of Invisalign^®^ orthodontic aligners following exposure to cigarette smoke.

## Methods

### Sample size

The sample size calculation was performed using G*Power software (version 3.1, Düsseldorf, Germany), with the following parameters: alpha = 0.05, power = 80% (0.8), and effect size = 1.25 [15]. The effect size was derived from a previous study that assessed color change in aligners [16], as no direct prior data were available for surface roughness, bacterial adherence, or pH changes in similar conditions.

Although color change differs from roughness and bacterial adhesion, both are influenced by surface modifications, which justified using this reference to estimate an appropriate sample size. To ensure statistical robustness, the calculated sample size was rounded up to 12 samples per group for the present study, allowing for a reliable comparison of bacterial adhesion and surface alterations after cigarette smoke exposure.

## Specimen preparation

With 12 samples per group, it was necessary to divide each surface for its respective analyses (Table 1), the specimens were divided and randomly assigned to experimental groups. Randomization was conducted using a computer-generated sequence to ensure unbiased allocation of specimens across groups. Specimens were randomly allocated into: growth control (G1), sterility control (G2), saliva (G3), and smoke (G4) groups. Additionally, linear roughness (Ra) and average depth (Rz) were made before (T0) and after (T1) exposure to saliva (G3.1) and smoke (G4.1), and bacterial adhesion and volumetric surface roughness (Sa) was evaluated with non-contact 3D profilometry, before (T0) and after (T1) exposure to growth control (G1), sterility control (G2), saliva (G3.2) and smoke (G4.2) groups.

**Table 1:** Distribution of the sample regarding aligner immersion and their respective characteristics for each analysis

Groups	Acronym	*n*	Definition	Analysis
Growth control	G1	12 flat aligner surfaces of 1 cm² each	Bacterial suspension prepared in 2% sucrose BHI broth.	Bacterial adhesion; pH; and Volumetric roughness (non-contact 3D profilometer)
Sterility control	G2	12 flat aligner surfaces of 1 cm² each	2% sucrose BHI broth	
Saliva	G3.1	12 vestibular surfaces of upper central incisors	Artificial saliva bath at 37 °C for 15 days	Surface roughness (digital roughness meters)
	G3.2	12 flat aligner surfaces of 1 cm² each		Bacterial adhesion; pH; and Volumetric roughness (non-contact 3D profilometer)
Smoke	G4.1	12 vestibular surfaces of upper central incisors	Artificial saliva bath at 37 °C for 15 days and exposed to 21 cigarette smoke cycles (10 cigarettes/cycle)	Surface roughness (digital roughness meters)
	G4.2	12 flat aligner surfaces of 1 cm² each		Bacterial adhesion; Volumetric roughness (non-contact 3D profilometer)

First, a total of 24 Invisalign^®^ samples from the upper central incisor region (tooth 11) were extracted from transparent Invisalign^®^ aligners (Align Technology, Santa Clara, California, USA), using sterile scissors, ligature cutter pliers, and procedural gloves. To assess surface roughness, it was necessary to trim only the vestibular surface of the upper central incisor to obtain a convenient sample piece for analysis. Surfaces with attachments (protrusions on the plastic material) were not selected. The shape and area of the trimmed surface of the aligners were standardized to ensure consistency across all samples. Each piece was precisely cut to a uniform size of 1 mm² x 1 mm², ensuring that all samples had the same dimensions. This standardization minimizes variability and allows for accurate comparisons. Since a larger surface area could provide more space for microorganism growth, maintaining a consistent size was essential for reliable results.

The area of choice for each tooth 11 was based on it being flat, absent of crowding or attachments, and coming from a healthy tooth, avoiding any kind of bias on the surface of the aligner. Thus, to assess bacterial adhesion, pH and volumetric roughness after experimental protocol, flat surfaces were cut from the vestibular surfaces of upper central incisors. A total of 72 specimens were needed, 12 per group for each analysis.

All samples were kept immersed in artificial saliva at 37 °C to mimic oral conditions under constant agitation, within an incubator (model 002 CB, Fanem, São Paulo/SP, Brazil) for 15 days, with saliva replacement performed three times a day. Regarding the aqueous medium, simulation was achieved by immersing the samples in 120 mL of artificial saliva (0.067% sodium chloride, 0.5% natrosol, 0.05% sodium benzoate, 2.4% sorbitol, deionized water qsp 100%, pH neutral).

## Exposure to cigarette smoke

The smoke exposure protocol was adapted from the methods described by Le Mesurier et al. [17] and Mathias et al. [18]. The specimens from the smoke group (G4.1 and G4.2) underwent a smoke assay to subsequently assess bacterial adhesion. For this experimental protocol, both the smoke and saliva experimental groups were exposed to artificial saliva under the same conditions for an equal period (15 days), with the difference that for the smoke group, exposure to artificial saliva also occurred after the completion of the cigarette smoke exposure cycles.

**Fig. 1:**
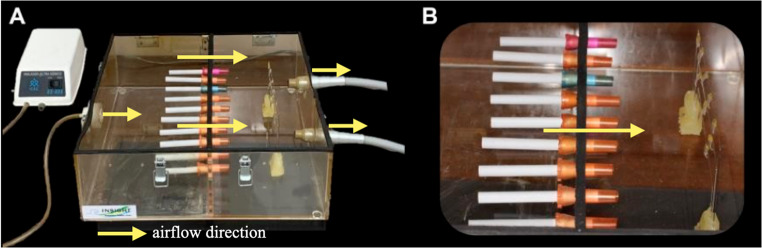
Acrylic device for cigarette smoke exposure. Representation of the custom-built acrylic chamber used to expose orthodontic aligner samples to cigarette smoke. (**A**) External view of the device showing the aligners positioned on metallic supports inside the chamber. (**B**) Internal view showing cigarettes positioned in the perforated partition and the sample platform inside the exposure chamber. Yellow arrows indicate airflow direction

**Fig. 2:**
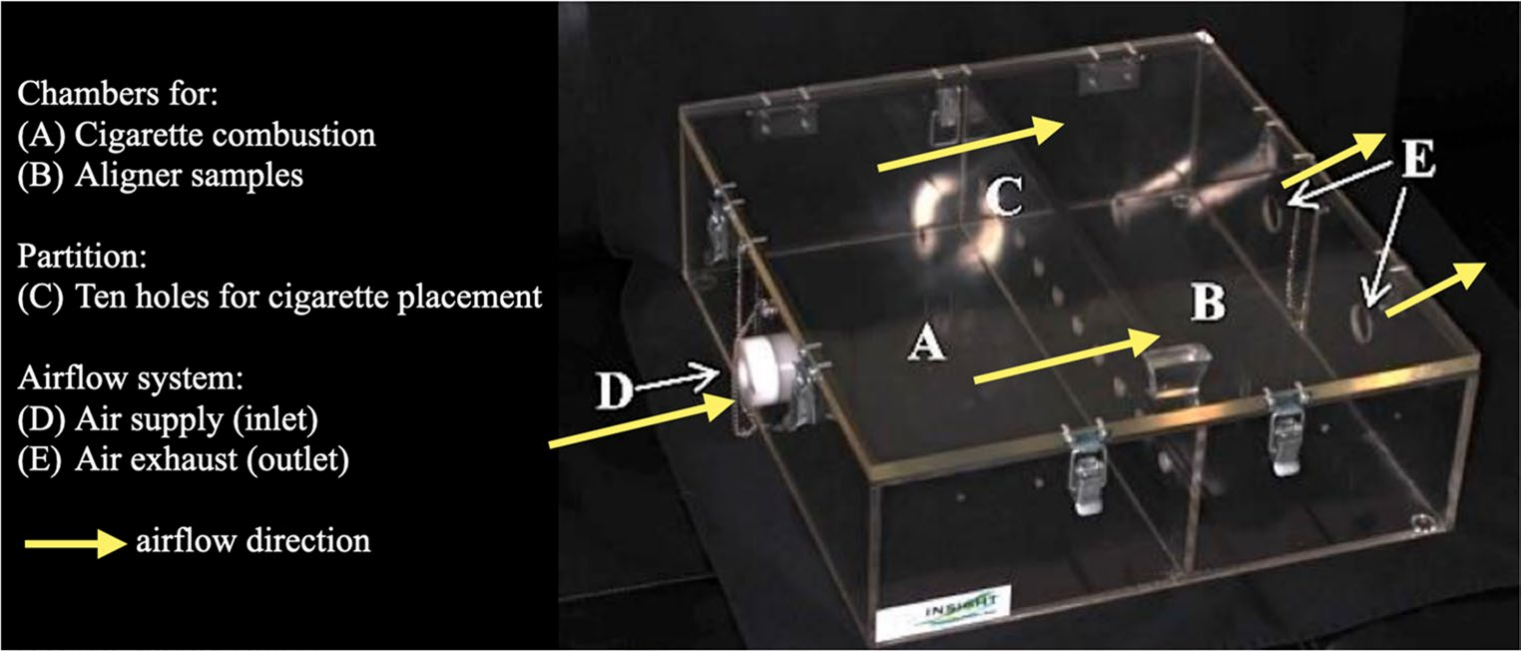
Detailed smoke exposure apparatus. Acrylic device consisting of two chambers: (**A**) chamber for cigarette combustion, and (**B**) chamber containing aligner samples. The chambers are separated by a partition with ten holes (**C**) for cigarette placement. Air is supplied through the inlet (**D**) and exhausted through two outlet holes (**E**), creating negative pressure that directs cigarette smoke through the partition toward the samples. Yellow arrows indicate airflow direction

**Fig. 3:**
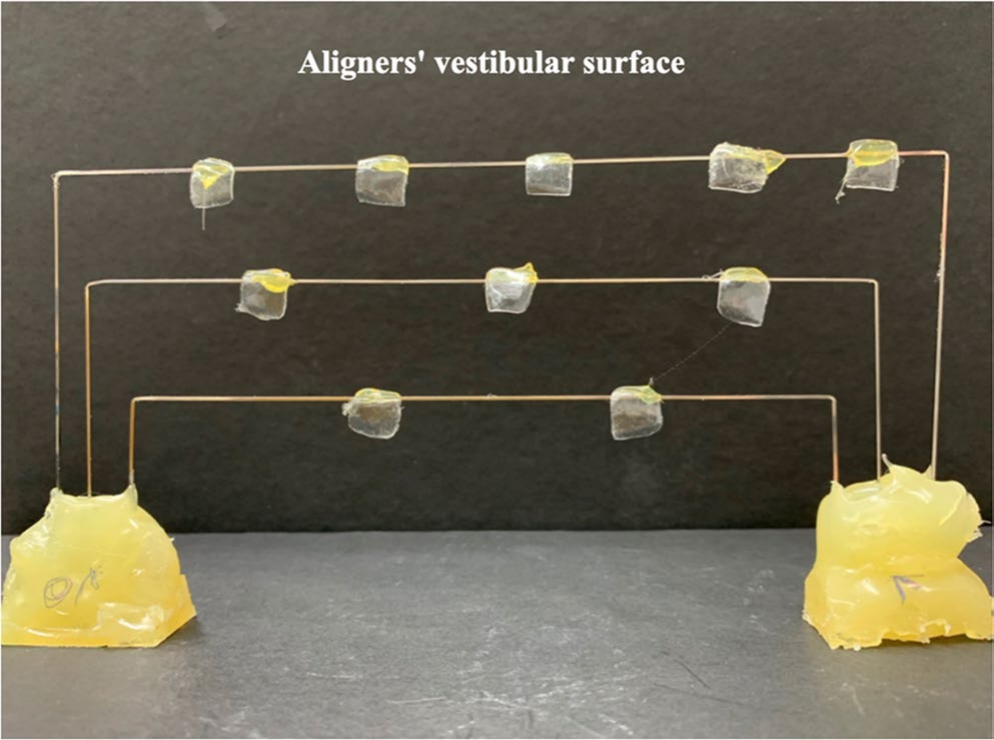
Sample positioning during smoke exposure. Samples were fixed by their lingual surfaces to steel wire supports, leaving the vestibular surfaces exposed to cigarette smoke. Note: Only a subset of specimens is shown for clarity; each group included 12 samples

A custom acrylic device was fabricated specifically for this study. The base consisted of self-polymerizing acrylic resin (JET – Classic, Campo Limpo Paulista, Brazil) with three embedded rectangular steel wire segments (0.018 × 0.025”, Morelli, Sorocaba, São Paulo, Brazil) (Fig. 1a). Cigarettes (Rothmans Blue, London, UK, imported by BAT Brazil) were placed in the holes of the divider, and after being lit, the device was sealed (Fig. 1b). Smoke generated from the burning cigarettes was directed toward the specimens in one of the chambers through a negative-pressure system. Pre-lit cigarettes were mounted on disposable plastic syringe protectors, with the filter side facing chamber B (Fig. 2), where the samples were placed (Fig. 3). Chamber A received external airflow from a compressor (US-800 Air, ICEL, Brazil) connected to the inlet (hole D) to maintain a constant air stream. Two suction devices attached to the outlets of chamber B (holes E) created negative pressure, forcing the smoke to pass through the cigarette filters and across the aligner surfaces.

Each cycle of 10 cigarettes burned for approximately 60 s and was replaced simultaneously to avoid opening the acrylic device frequently, a total of 21 cycles was completed to achieve a cumulative exposure level that simulates prolonged contact with cigarette smoke, ensuring sufficient deposition of smoke residues on the samples for analysis (10 cigarettes per cycle) [17, 18]. The choice of 10 cigarettes per cycle was made to ensure a controlled and uniform exposure while minimizing the need to frequently open the acrylic device, which could alter the smoke concentration inside. Each cycle lasted approximately 60 s, allowing all 10 cigarettes to burn simultaneously before being replaced.

The samples from the G4 group were detached from the wires using a wire-cutting pliers, and the hot glue was gently removed from each specimen using a n^o^.12 scalpel blade, to avoid damaging the samples previously positioned in hot glue. Following removal, the samples were placed in an ultrasonic bath (Cristófolli, Campo Mourão, Paraná, Brazil) with distilled water for 30 s to remove any excess smoke residue that may have been impregnated on the surface of the aligners.

## Surface roughness analysis

**Fig. 4:**
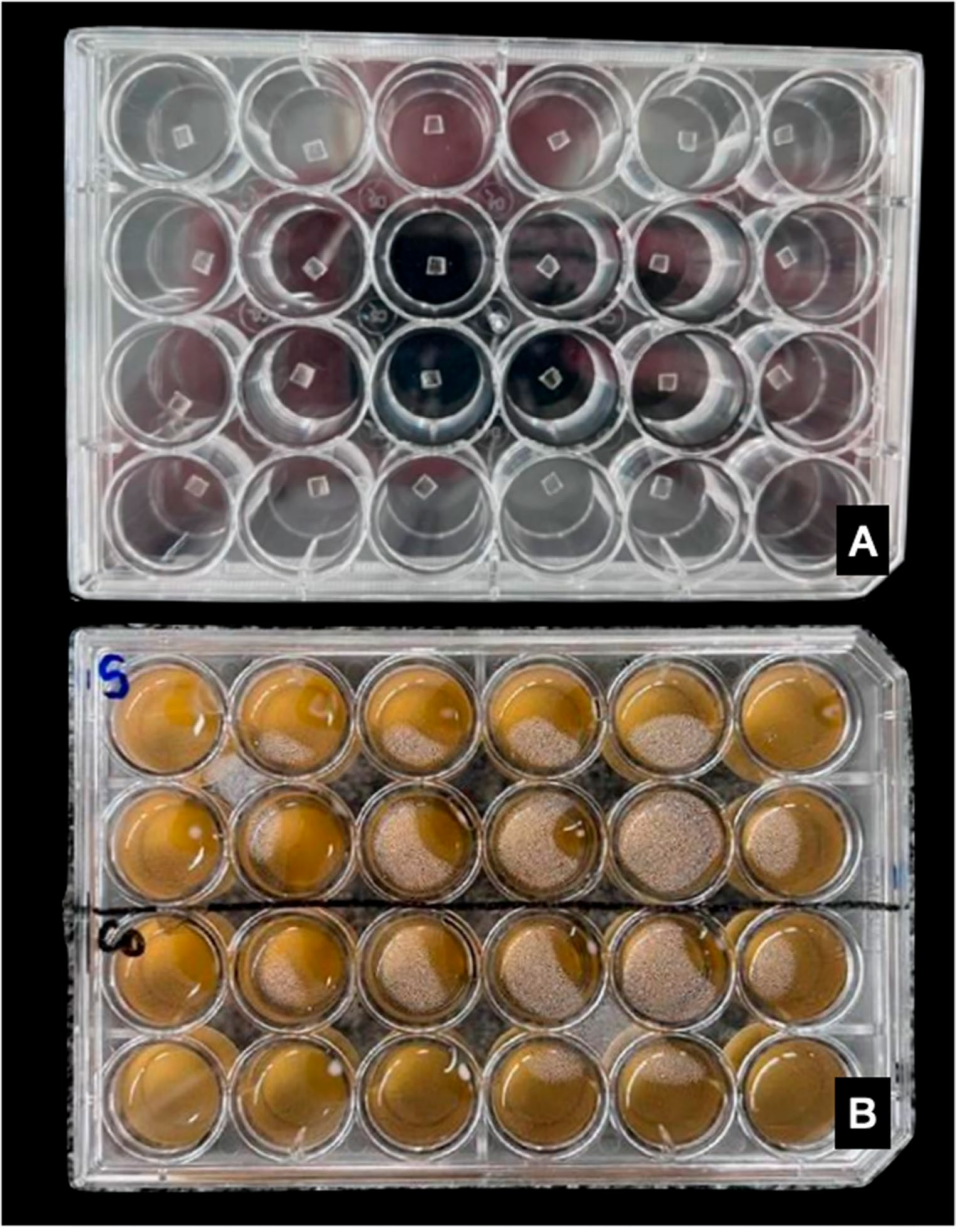
Samples not exposed (**A**) and after exposure to cigarette smoke (**B**)

Surface roughness was evaluated using a digital contact roughness meter (Mitutoyo SJ-310) according to ISO 4287 − 1997 standards. Each specimen was fixed to the test table of the device, and roughness was measured in the middle region of the vestibular surface. The parameters measured were average roughness (Ra) and mean depth of roughness (Rz) of the groups G3.1 and G4.1 (Fig. 4), measurements were taken before exposure to saliva and cigarette smoke (T0) with those taken after exposure solely to saliva, as well as with groups exposed to cigarette smoke (T1).

## Microbiological analyses

Initially, the strains of *Streptococcus mutans* (ATCC 25175), *Lactobacillus casei* (ATCC 393), and *Candida albicans* (ATCC 90028) were reactivated using a standardized bacterial suspension [19], which was transferred to 2% sucrose BHI broth (pH = 7.10). The microbial strains selected for this study were chosen due to their well-documented relevance in the development of dental biofilms and their association with caries pathogenesis. The inclusion of these three microorganisms aimed to simulate a clinically relevant multispecies biofilm, especially reflective of conditions in patients with compromised oral hygiene or tobacco use. The strains were verified based on the optical density of 0.08 to 0.13 nm, considering the wavelength at 625 nm (bacteria) and 530 nm (fungi) using a spectrophotometer. After reactivation, the strains were separated after subsequent microbiological analysis.

After exposure to cigarette smoke, the experimental groups smoke (exposed to smoke, G4.1 and G4.2) and saliva (not exposed to smoke, G3.1 and G3.2), and two control groups (growth, G1 and sterility, G2) were considered for microbiological analyses, with 12 samples per group. The samples were randomly divided into these four groups, where the growth group (G1) consisted of a bacterial suspension [multispecies biofilm of *S. mutans* (ATCC 25175), *L. casei* (ATCC 393), and *C. albicans* (ATCC 90028)] prepared in 2% sucrose BHI broth was used. Therefore, the sterility group (G2) was immersed in 2% sucrose BHI broth without the presence of multispecies biofilm. The saliva group (G3.2) is characterized by samples that were immersed only in artificial saliva, while the smoke group (G4.2) also had the immersion in artificial saliva but samples were also exposed to 21 cycles of cigarette smoke.

**Fig. 5:**
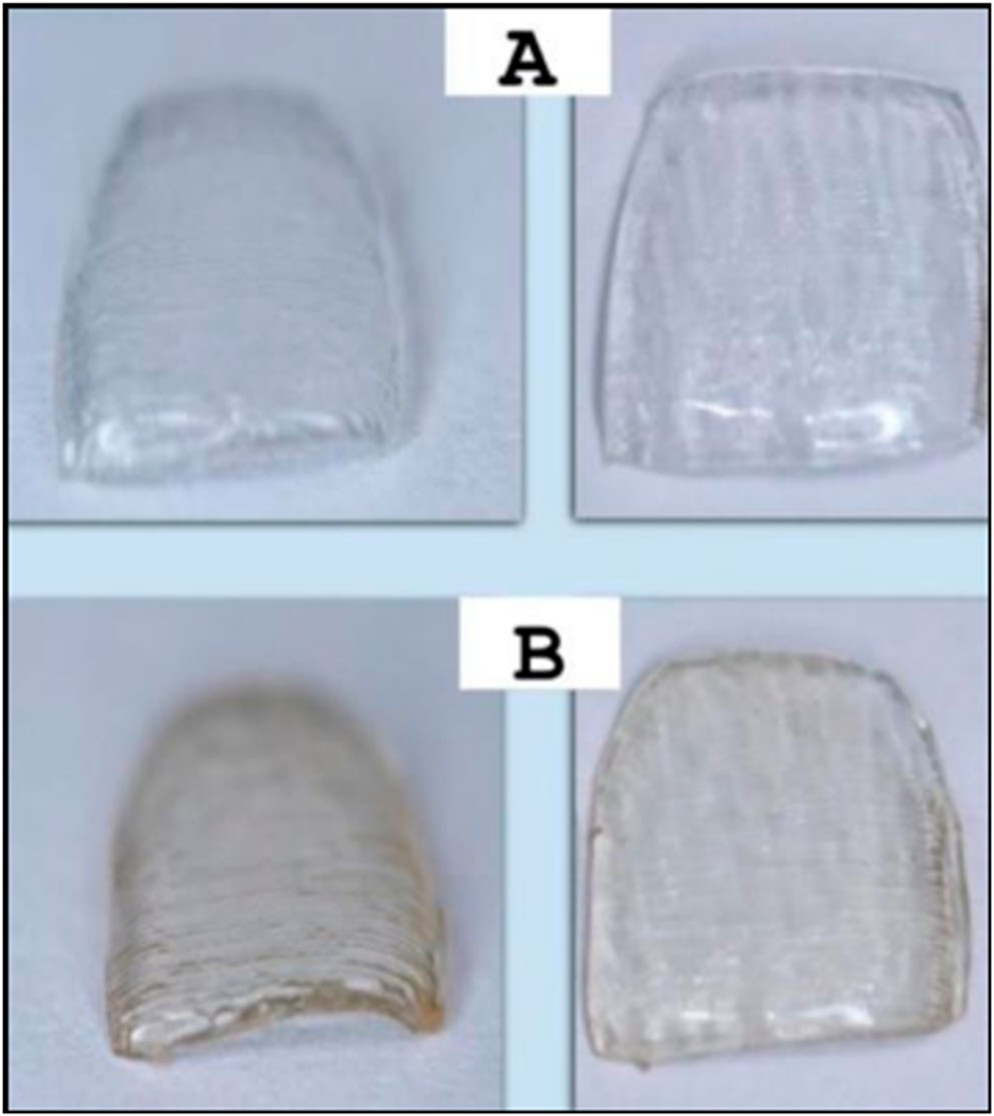
Distribution of samples in a polystyrene culture plate with 24 wells, with 12 aligners surfaces per group (**A**); Samples immersed in multispecies biofilm (**B**)

All the samples were arranged in four polystyrene culture plates with 24 wells (model K12-024, Kasvi^®^, São José do Pinhais, BRA), illustrated in Fig. 5a, followed by sterilization using ultraviolet light (40 W) [20, 21] in a laminar flow hood, with an exposure time of 1 h. Then, all the samples were previously immersed in 2 mL of artificial saliva [22] at 37 °C again for 1 h in a microbiological incubator before the immersion of bacterial, to eliminate any surface-related residues. After this period, the saliva was removed, 2 mL of the mixed inoculum (5 × 10^5^ CFU/mL final concentration) was inserted for incubation at 37 °C for 24 h [19].

After 24 h of initial biofilm formation (Fig. 5b), the culture medium (BHI) was removed using a pipettor, and fresh culture medium (2 mL of BHI) was added for further incubation at 37 °C for an additional 24 h. The total duration of the analysis was five days because measurements were taken once after each immersion period. Since the samples were immersed for four days, the analysis covered five time points in total, including the initial measurement before any exposure to assess baseline conditions. The culture medium was changed once daily throughout the five-day experiment, specifically at 24, 48, 72, and 96 h. This approach allowed for tracking changes over time, from the start of the experiment through the full immersion period.

Only after 24 h from the start of the biofilm formation, the culture medium of each sample was collected for subsequent pH evaluation [21]. The acidogenicity of the biofilms was assessed by measuring the pH of the culture medium during the periods of 24, 48, 72, and 96 h of the bacterial adhesion test. This evaluation was performed in duplicate using a microelectrode (PHOX^®^, Colombo, BRA).

### Colony forming unit (CFU/mL) count

After 96 h, the specimens (G1, G2, G3.2 and G4.2) were individually transferred to 1 mL sterile tubes (Eppendorf North America, Inc., Connecticut, USA) containing 1 mL of saline solution (NaCl). The specimens were vortexed for 1 minute to detach the biofilm, and aliquots of suspended biofilm were used for quantification of viable microorganisms. For this quantification, the biofilm suspension was serially diluted (10^1^ to 10^6^), from which 50 µl aliquots were collected from dilutions 10^4^ and 10^6^ and plated on BHI agar and incubated at 37 °C for 24 h. After this period, the colony-forming units (CFU/mL) were counted, with two researchers performing the counting independently, and in case of discrepancy between the analyses, a third evaluator would also perform the counting.

## Volumetric roughness analysis

Volumetric surface roughness (Sa) was evaluated after the bacterial adhesion protocol using non-contact 3D profilometry (Nanovea PS50 Optical, NANOVEA^®^, Irvine, USA). For this purpose, a standardized evaluation area of 1 mm^2^ on the surface of the aligners, corresponding to the sample size, was used. The mean values of three measurements of volumetric roughness (Sa) (250 µm^2^) were obtained for each specimen (G1, G2, G3.2 and G4.2).

### Statistical analysis

Statistical analysis was performed using SPSS v.26 software (IBM Corp., Armonk, NY, USA). Data distribution was assessed using the Shapiro–Wilk test. For data meeting the assumptions of normality and homogeneity of variance, an independent-sample t-test was applied to compare roughness parameters (Ra and Rz) between G3.1 and G4.1, and a paired t-test was used to evaluate changes within groups over time (T0 vs. T1). When normality or homogeneity assumptions were not met, non-parametric tests were employed: Kruskal–Wallis for overall comparisons and Mann–Whitney for pairwise analyses. These were used, in particular, for pH, colony-forming unit (CFU/mL) data, and surface roughness (Sa) after bacterial adhesion among G1, G2, G3.2, and G4.2.

## Results

Table 2 presents the descriptive and comparative statistics for the Ra and Rz surface roughness parameters. When comparing G3.1 and G4.1 at T0, there was no difference in either Ra or Rz (*p* = 0.952 and *p* = 0.514, respectively), indicating that both groups exhibited similar surface roughness at the beginning of the experiment. However, when comparing G3.1 and G4.1 at T1, there was a significant difference (*p* = 0.046) for Ra (G3.1: 0.122 ± 0.002; G4.1: 0.139 ± 0.007), with higher values observed in G4.1. Similarly, for Rz, there was a significant difference (*p* = 0.002) between G3.1 (0.616 ± 0.021) and G4.1 (0.741 ± 0.026) at T1, with higher values found in G4.1.

**Table 2:** Descriptive data and comparison of roughness parameters between the control saliva group (G3.1) and experimental smoke (G4.1) groups. Roughness parameters (µm)

		G3.1	G4.1	*p*-value
	T0	0.124 ± 0.003	0.123 ± 0.005	0.952
Ra	T1	0.122 ± 0.002	0.139 ± 0.007	0.046*
	p-value	0.807	0.003*	
	T0	0.586 ± 0.025	0.612 ± 0.030	0.514
Rz	T1	0.616 ± 0.021	0.741 ± 0.026	0.002*
	p-value	0.231	0.014*	

Legend: T0 = before exposure to saliva and cigarette smoke; T1 = after exposure to saliva and cigarette smoke; *Significant statistical difference (*p* < 0.05)

In G3.1, there was no statistically significant difference in Ra (*p* = 0.807) and Rz (*p* = 0.231) when comparing samples between T0 and T1, although Rz values increased, suggesting that artificial saliva may not significantly influence these parameters. Conversely, in G4.1, both Ra (*p* = 0.003) and Rz (*p* = 0.014) increased, indicating that cigarette smoke interferes with aligner surface roughness.

**Fig. 6:**
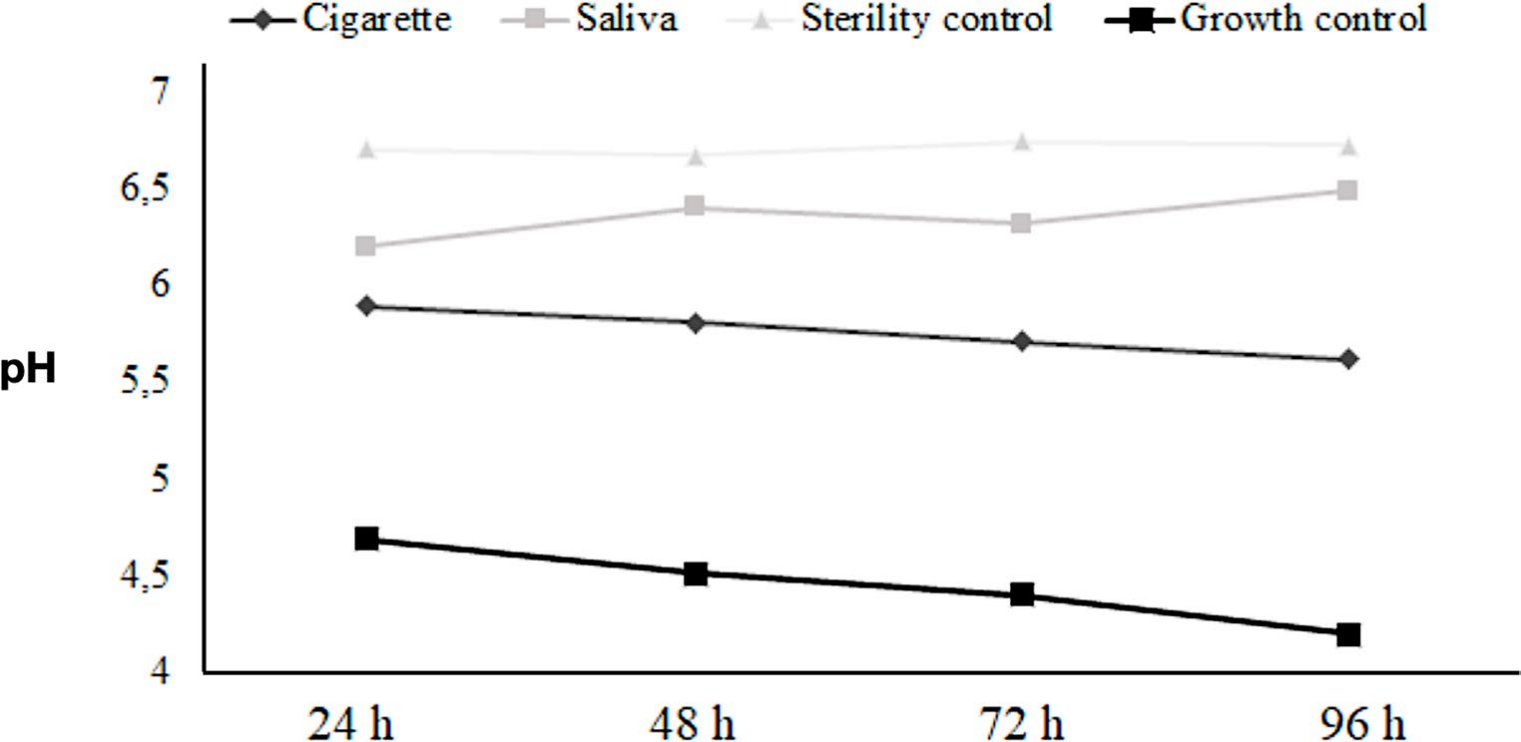
pH variation over 24, 48, 72, and 96 h

During the bacterial adhesion protocol, pH variations were analysed over 24, 48, 72, and 96-hour periods (Fig. 6). In the analysis of the first 24 h of the experiment, the growth control group (G1) showed the lowest pH, around 4.5. Similarly, the smoke group (G4.2) exhibited a low pH, around 5.7. Only these two groups showed a constant pH decrease throughout the experiment, reaching approximately pH = 4 (G1) and pH = 5.5 (G4.2). In contrast, the sterility control group (G2) maintained a stable pH over 96 h, around 6.7. Similarly, the saliva group (G3.2) had a pH of around 6.2 in the first 24 h, slightly increased to approximately 6.3 at 48 h, stabilized, and reached around 6.5 at 96 h. The G4.2 shows a slight decrease, while G3.2 shows an increase, suggesting that saliva promotes growth more effectively than smoke exposure inhibits it (*p* < 0.005). For temporal changes, 24 to 96 h, minor changes are observed in all conditions, with slight decreases in smoke (5.89 to 5.62) and growth control (4.69 to 4.2), and slight increases in saliva (6.2 to 6.49) and sterility control (6.7 to 6.84).

**Fig. 7:**
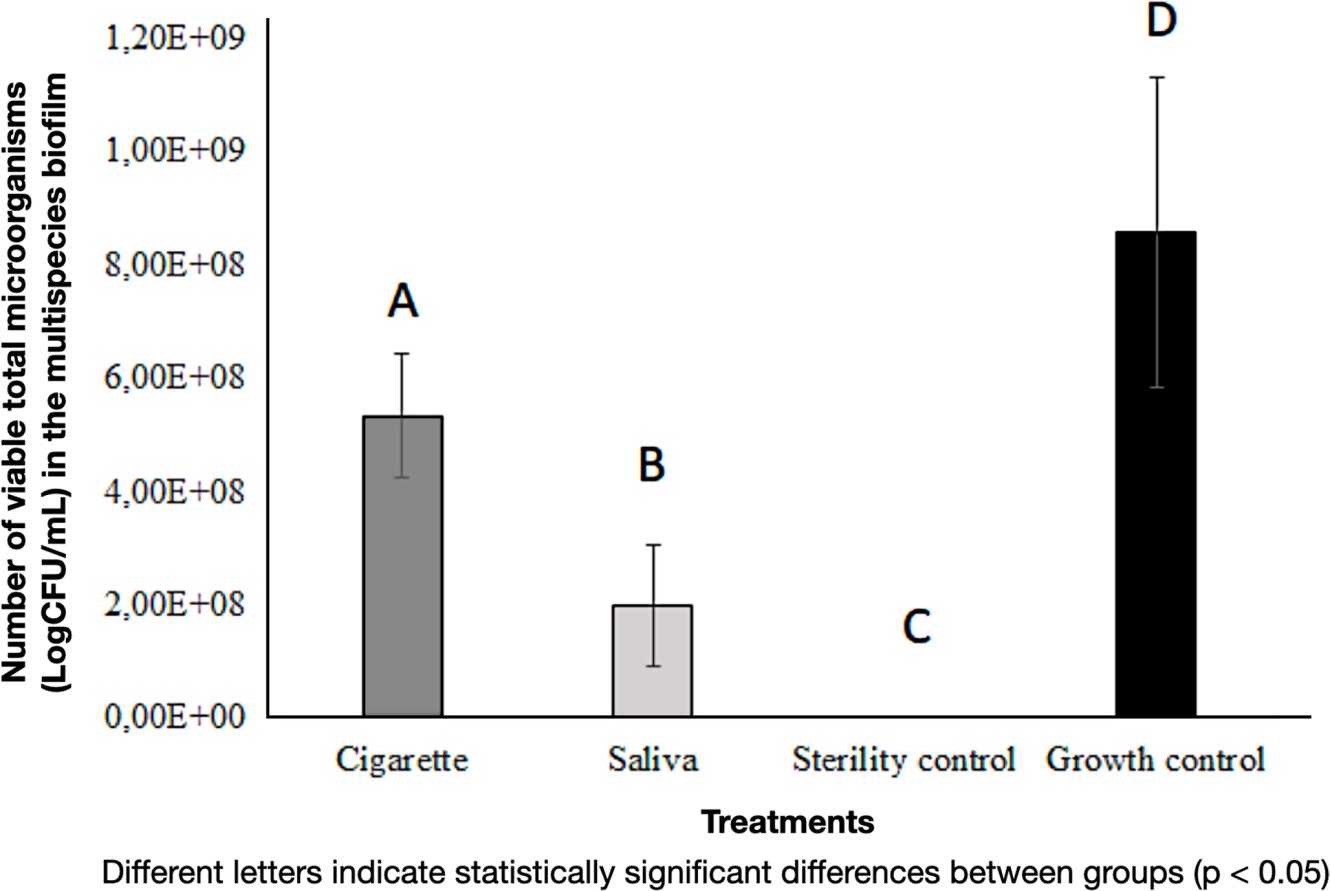
Quantification of total microorganisms in each group. Bars represent mean ± standard deviation. Different letters indicate statistically significant differences (*p* < 0.05) according to the Kruskal–Wallis and Mann–Whitney tests

Regarding the quantity of microorganisms in the culture medium (Fig. 7), it can be observed that the growth of microbial colonies was most significant in the growth control group (G1), composed of multispecies biofilm prepared in 2% sucrose BHI broth, while the sterility control group (G2) showed no growth. Compared to the saliva group (G3.2), the smoke group (G4.2) exhibited higher bacterial growth. The smoke group (5.33 × 10^8^) had more than double the CFU count of the saliva group (2 × 10^8^) (*p* < 0.005). The growth control had the highest CFU count (8.57 × 10^8^), significantly higher than the other groups (*p* < 0.005).

**Fig. 8:**
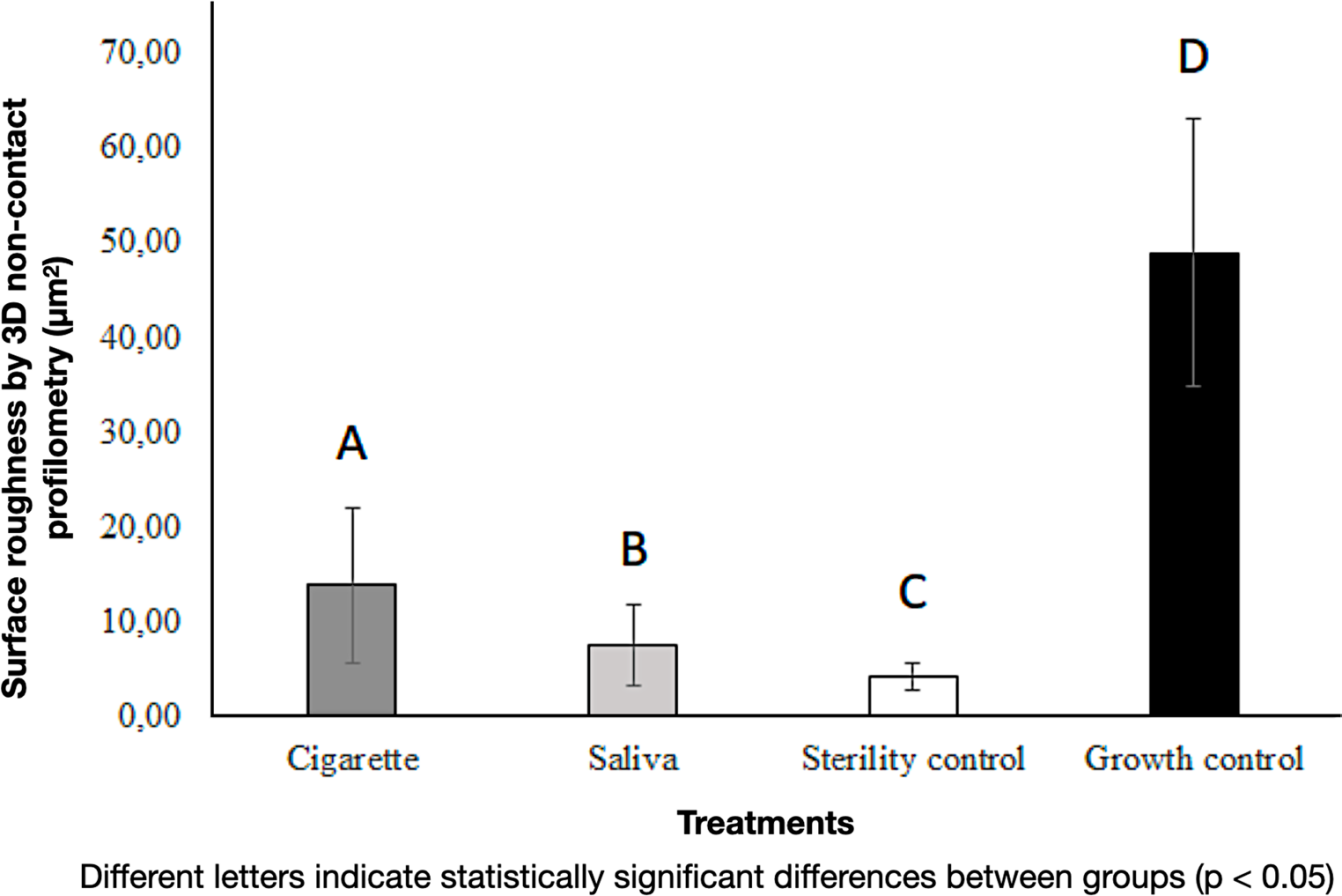
Surface roughness (Sa) in each group. Bars represent mean ± standard deviation. Different letters indicate statistically significant differences (*p* < 0.05) according to the Kruskal–Wallis and Mann–Whitney tests

Regarding the analysis of non-contact 3D profilometry (Fig. 8), it can be observed that the growth control group (G1) (48.97 ± 14.19) exhibited the highest surface roughness value (*p* < 0.005). Samples from the saliva group (G3.2) (7.60 ± 4.28), followed by samples from the smoke group (G4.2) (13.98 ± 8.18), showed the roughest surfaces in the experiment (*p* < 0.005). In this experiment, the sterility control group (G2) (4.27 ± 1.14), which was not exposed to any bacterial suspension, exhibited the lowest roughness value.

## Discussion

This in vitro study analysed surface roughness, alterations in adherence and biofilm formation rates of *Streptococcus mutans*,* Candida albicans*,* and Lactobacillus casei* on Invisalign^®^ orthodontic aligner surfaces following cigarette smoke exposure, in addition to assessing changes in pH, and volumetric roughness during bacterial adhesion protocol.

Orthodontic aligners possess retentive surfaces serving as ideal substrates for bacterial biofilm formation [14, 23] and disrupting microbial homeostasis in the oral cavity [24]. Physical factors such as surface roughness and morphology, surface free energy, surface tension, hydrophobicity, and substance absorption affinity play significant roles on solid surfaces [25]. Experimental analyses [26, 27] have demonstrated the impact of cigarette smoke exposure on aligners. Initially, the surface of Invisalign^®^ aligners is designed to be smooth, minimizing bacterial adhesion. However, after exposure to cigarette smoke, our findings revealed a significant increase in surface roughness, which can facilitate bacterial and fungal colonization. This study is the first to establish a direct correlation between cigarette smoke exposure and the adherence and biofilm formation rates of *S. mutans* and *C. albicans* on Invisalign^®^ aligners, highlighting the potential oral health risks for smoking patients.

The surface topography of aligners is irregular [5, 28], even in aligners never exposed to the oral environment. Based on the results of the present study, there was no difference in Ra and Rz values for aligners exposed solely to artificial saliva. However, it is known that in vivo, various adversities inherent to the oral environment, such as enzyme actions, pH alterations, consumption of acidic beverages, and potential abrasive effects of masticatory movements, can exacerbate irregularities and surface heterogeneity, further contributing to increased material roughness.

The findings of this study suggest that cigarette smoke significantly affects Ra and Rz surface roughness parameters, corroborating with other findings where roughness parameters increased after oral use [11, 29]. These results, however, contrast with data from a previous study [6] where a decrease in roughness was observed after intraoral aligner use for 14 days. The difference in methodology proposed possibly explains the finding, as it may have induced a wear effect, resulting in subsequently smoother surfaces after use.

Isolated roughness parameters may not accurately reflect the true surface topography of a tested material. Therefore, employing various evaluation methods, such as scanning electron microscopy, optical profilometry and microscopy [29, 30], is essential for a more comprehensive understanding.

Recent systematic review and meta-analysis [9] on oral hygiene parameters revealed that orthodontic treatment with Invisalign^®^ without the use of any accessories or attachments is associated with lower biofilm scores compared to fixed appliances in non-smoking patients. The increase in polymer roughness can stimulate biofilm accumulation [31], aligners exposed to cigarette smoke may be more prone to biofilm accumulation and retention [23], raising questions about stricter oral hygiene maintenance [26], who are even more affected by bacterial retention [28].

Further in vivo and in vitro studies quantifying formed biofilm are necessary for categorical statements that cigarette smoke exposure increases Invisalign^®^ aligner surface roughness and, therefore, results in greater bacterial biofilm accumulation on its surface. In this in vitro study, differences in bacterial adherence, pH, and surface roughness were observed in Invisalign^®^ orthodontic aligner samples exposed to cigarette smoke, artificial saliva, and samples from control groups (sterility and growth). The sterility control group showed no microbial growth, indicating that laboratory working conditions were effective against bacterial contamination. Conversely, the group exposed to cigarette smoke showed higher bacterial growth compared to the group exposed only to saliva. This highlights that bacteria have greater ease of colonizing aligners in smoking patients, increasing the risk of oral infections and health problems [23, 32].

The microscopic findings revealed irregularities on orthodontic aligner surfaces, even in the sterility control group, which may result from the aligner manufacturing process. Identifying these irregularities, as surface roughness in plastic material may favour the formation of surface porosities, increasing bacteria retention and biofilm accumulation, which in turn enhances acquired film and bacterial plaque formation.

The growth control and smoke group exhibited the roughest surfaces in the study compared to the other groups, while samples from the sterility control group presented the least rough surface. Meanwhile, bacterial quantification analysis on the sterility control group aligner surfaces showed no statistically significant differences, as the presence of bacteria was close to zero. In this perspective, it is necessary to consider that, as reported in other studies in the literature, different factors can influence bacterial biofilm formation on aligner surfaces. These factors include prolonged aligner use [11], acidic pH of the oral environment [27], poor oral hygiene by the patient [33], and inadequate cleaning of plastic aligners [25, 30, 34].

Although the manufacturer recommends not wearing aligners while smoking, it is well known that there is a demand from adult patients who smoke and do not always follow these guidelines [35]. Exposure of Invisalign^®^ orthodontic aligners to cigarette smoke had a significant impact on bacterial adherence, pH, and surface roughness. The pH measurement presented two evaluation thresholds, minimum and maximum, represented by saliva group (G3.2) and smoke group (G4.2), respectively. The sterility control group (G2) maintained the highest pH throughout the experiment, while the growth control group (G1) exhibited a constant decrease.

These findings align with recent evidence in the literature, including a 2024 systematic review on home biofilm management during invisible orthodontic treatment [34]. Although mechanical methods alone appear more effective than chemical ones, the review highlights that the best outcomes are achieved through a combination of both. This supports the clinical recommendation that patients undergoing treatment with aligners, especially smokers or individuals at higher risk of biofilm formation, adopt a synergistic hygiene approach incorporating both mechanical and chemical cleansing agents. Given the increased bacterial adherence and surface roughness observed following cigarette smoke exposure in the present study, as well as the alterations in optical properties and color stability reported by Minervini et al. [35], reinforcing comprehensive hygiene protocols becomes even more critical to mitigate potential oral health risks and preserve aligner integrity.

This study has some limitations that should be acknowledged. First, the sample size calculation was based on an effect size obtained from a previous investigation on color change in thermoplastic aligners, as no specific data on surface roughness were available at the time of study design. Although this surrogate parameter may introduce bias, the number of specimens was sufficient to detect statistically significant differences in surface roughness (Ra, Rz, Sa) and microbiological outcomes. In addition, the cigarette smoke exposure protocol adopted here, 21 cycles of 10 cigarettes, was adapted from previously published in vitro models intended to accelerate the cumulative effects of smoke on dental materials. While this approach enabled relevant surface and microbiological changes to be observed within a feasible experimental timeframe, it does not replicate real-world smoking patterns. Therefore, the findings should be interpreted within the context of an accelerated laboratory exposure model rather than as a direct reflection of daily smoking habits. Future research should aim to establish reference effect sizes for roughness changes in aligner materials and to refine exposure protocols that more closely mimic actual patient behaviors.

Janson et al. [36] recently demonstrated that exposure of universal composite resins to common staining solutions (coffee, red wine, matcha tea, and artificial saliva) resulted in significant color changes and alterations in surface roughness, although no correlation was observed between roughness and the degree of discoloration. Their results highlight that optical changes and surface degradation do not necessarily progress in parallel, emphasizing the multifactorial nature of material deterioration under environmental challenges. In line with these findings, the present study shows that cigarette smoke exposure may similarly accelerate degradation in aligner polymers, thereby creating more favorable conditions for biofilm retention. Together, these results suggest that both surface alterations and intrinsic compositional features of esthetic orthodontic materials play a crucial role in microbial adhesion. Moreover, the lack of a direct correspondence between surface topography and other outcomes (such as discoloration or biofilm accumulation) underscores the importance of comprehensive assessments that combine physical, optical, and biological parameters, as performed in the current investigation.

In conclusion, exposure to cigarette smoke not only influences the increase in bacterial quantity but also increases acidification of the oral environment and rougher surfaces on orthodontic aligners in this in vitro study. Therefore, even if aligners are removed while smoking, bacterial colonization may still be a concern due to residual effects. Smoke-related compounds can persist in the oral cavity, coating the teeth, gums, and saliva, potentially affecting the aligners once they are reinserted. Additionally, smoking alters the oral microbiome and reduces salivary flow, creating a more favorable environment for bacterial growth, further reinforcing the clinical relevance of our findings.

## Conclusions

Cigarette smoke exposure increases aligner surface roughness and enhances bacterial biofilm formation, accompanied by greater acidification of the oral pH. These findings reinforce the need for strict hygiene protocols and smoking cessation counseling for aligner-wearing patients.

Clinically, practitioners should be aware that smoke exposure compromises aligner integrity and favors microbial colonization, underscoring the importance of reinforcing cleaning measures and confirming these effects through in vivo studies.

## Data Availability

No datasets were generated or analysed during the current study.
